# Bridging the Gap between Theory and Practice in Integrated Care: The Case of the Diabetic Foot Pathway in Tuscany

**DOI:** 10.5334/ijic.1991

**Published:** 2016-05-24

**Authors:** Sabina Nuti, Barbara Bini, Tommaso Grillo Ruggieri, Alberto Piaggesi, Lucia Ricci

**Affiliations:** Management and Health Laboratory, Institute of Management, Scuola Superiore Sant’Anna, Via San Zeno 2 – 56127, Pisa, Italy; Diabetic Foot Section, Department of Medicine, Teaching Hospital of Pisa, Pisa, Italy; Division of Diabetology, San Donato Hospital, Arezzo, Italy

**Keywords:** diabetes, diabetic foot, geographic variation, performance evaluation, benchmarking, sentinel events, engagement

## Abstract

**Introduction and Background:**

As diabetic foot (DF) care benefits from integration, monitoring geographic variations in lower limb Major Amputation rate enables to highlight potential lack of Integrated Care. In Tuscany (Italy), these DF outcomes were good on average but they varied within the region. In order to stimulate an improvement process towards integration, the project aimed to shift health professionals’ focus on the geographic variation issue, promote the Population Medicine approach, and engage professionals in a community of practice.

**Method:**

Three strategies were thus carried out: the use of a transparent performance evaluation system based on benchmarking; the use of patient stories and benchmarking analyses on outcomes, service utilization and costs that cross-checked delivery- and population-based perspectives; the establishment of a stable community of professionals to discuss data and practices.

**Results:**

The project enabled professionals to shift their focus on geographic variation and to a joint accountability on outcomes and costs for the entire patient pathways. Organizational best practices and gaps in integration were identified and improvement actions towards Integrated Care were implemented.

**Conclusion and Discussion:**

For the specific category of care pathways whose geographic variation is related to a lack of Integrated Care, a comprehensive strategy to improve outcomes and reduce equity gaps by diffusing integration should be carried out.

## Introduction

Although many countries aim for more Integrated Care within and across institutional boundaries as a means of developing more cost-effective health services [1,2,3,4], this often fails because of established working patterns.

As good outcomes for the Diabetic Foot (DF) pathway depend on the involvement of several clinicians across different care settings and institutions, this pathway is an exemplar of the benefits of integration. Despite international recommendations to diffuse multi-disciplinary teams and integrated paths in order to improve DF outcomes [5,6,7,8,9,10,11,12,13], various factors are needed to achieve Integrated Care in the pathway organization.

This study contributes to the debate on how to implement Integrated Care [14,15,16] and bind together the research fields on the relevance of the epidemiological surveillance of medical care [17,18,19,20,21,22,23,24] and the public reporting of performance [25,26,27,28,29,30].

The paper describes the successful implementation of a combination of strategies aimed at spreading Integrated Care within the DF pathway in Tuscany (central Italy). The objectives linked to specific strategies are to:

– encourage clinicians to focus on the geographic variation issue;
– spread the Population Medicine perspective;
– engage clinicians in a stable community of practice in order to identify gaps in integration and practical models of Integrated Care [31,32].

## Background

### The Diabetic Foot Pathway in Tuscany

Tuscany is a region in central Italy with approximately 3.7 million inhabitants. Its regional healthcare system follows the Beveridge model and provides universal coverage with an annual public budget of €6.6 billion.

Twelve local health authorities (LHAs) are responsible for organizing and providing comprehensive healthcare services for an average of approximately 300,000 inhabitants. Hospital care is provided by general hospitals led by LHAs and three teaching hospitals (THs), which are independent health authorities (HAs) without a specific geographic catchment area and regional referral centres for complex care.

Since 2004, the regional government in Tuscany has entrusted the “Management and Health Laboratory” (MeS-Lab) of the Scuola Superiore Sant’Anna University with the design and management of a multi-dimensional healthcare performance evaluation system (PES) [33,34,35]. Using systematic benchmarking, the PES compares the results of the twelve LHAs and the three THs in Tuscany considering 130 indicators. Since 2008, the PES has also included other Italian regions for systematic inter- and intra-regional comparisons [35]. Data are published on http://performance.sssup.it.

**Figure 1:**
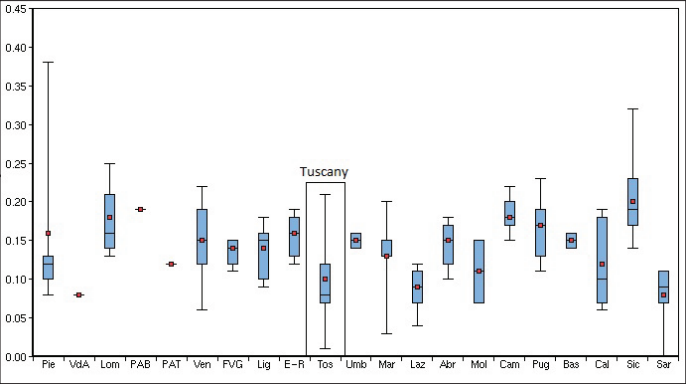
Age- and gender-adjusted hospitalization rates for diabetes-related lower limb amputations (major and minor) per 100,000 residents in the Italian Regions and Provinces – 2012. Source: National Outcome Evaluation Program – National Agency for Regional Health Services.

In 2012, the Tuscany Regional Government entrusted the MeS-Lab to design and coordinate an action plan to reduce the variation in DF outcomes by disseminating Integrated Care. The Italian National Outcomes Evaluation Programme [36] showed in 2012 that DF outcomes vary across regions and local areas (Figure 1).

Despite Tuscany performed well compared to the other regions, there were considerable internal variations among its Provinces. This variability across and within regions suggested potential differences in implementing international guidelines, leading to unwarranted geographic variations, i.e. variations not dictated by the needs of populations and patient preferences [17,18,19,20,21,22,23,24]. Such variations ought to be remedied within those healthcare systems aimed at reducing equity gaps (as in the case of the Italian Beveridge model) [21,23,24].

The Regional Government also chose the DF pathway because of the increasing:

– Incidence of DF complications, i.e. the first cause of lower-limb amputations in industrialized countries [9,37];
– social and financial costs of DF patients who have had major amputations. DF care is associated with high additional costs of diabetes care for patients and healthcare systems [38,39].
– the importance of assessing the overall quality of care in terms of the rate of diabetes-related lower limb major amputation, which is an indicator of poor quality and poor coordination within the overall service chain, which should trigger further investigations [40,41].

### The definition of Integrated Care and the Diabetic Foot Pathway

Integrated Care covers a rich conceptual framework with many theoretical definitions [1,2,3,4,42,43,44,45,46,47,48,49,50,51], and different categories, breadth and degree.

Kodner [3] proposed six categories of integration:

– functional integration as the coordination between back-office and support functions across all units;
– organizational integration as the relationships between healthcare organizations;
– professional integration as the provider relationships within and between organizations;
– service or clinical integration as the coordination of services and the integration of care in a single process across time, place and discipline;
– normative integration as the shared mission, work values and organizational/professional culture;
– systemic integration as the alignment of policies and incentives at the organizational level.

Valentijn and colleagues described also three different levels of integration [46]: macro-level integration operates across sectors; meso-level integration operates both within and between organizations in order to diffuse collective actions across the entire care continuum overcoming cultural, professional and bureaucratic boundaries; micro-level integration operates between clinicians and the individual patient in order to enforce the continuity of care and meet patient needs, regardless of specific organizational functions or structures.

In terms of breadth, integration between different organizations is known as vertical integration and the coordination of similar units or settings of care as horizontal integration [3,52].

Leutz described three configurations based on the intensity (i.e. degree) of the connections between organizations and units [50]: linkage, coordination and full integration. Linkage promotes the continuity of care for the individual patient through existing and autonomous organizations with adequate communication and referrals to match services with patients’ needs. Coordination *‘identifies points of friction, confusion, or discontinuity between systems and establishes structures and processes to solve these problems’* [50, p. 85] (e.g. increasing information-sharing, managing transition of care between settings). Full integration operates when a new accountable entity is established by pooling resources, rather than improving the coordination of the existing units.

The DF pathway involves all the settings of care, from community-based nursing clinics and primary care units, to highly-complex hospital departments (e.g. vascular surgery), often belonging to different organizations and institutions.

Based on the organization of the Tuscany Healthcare System and the conceptual framework of integration, in this study we identify the need for integration between different:

– units within the same organization (e.g. departments within a hospital);
– care settings (e.g. LHA primary care units and LHA-led hospitals)
– institutions (e.g. LHA nursing clinics and Teaching Hospitals).

With particular regard to normative integration, we considered the Population Medicine approach as the set of values that should inform professional behaviors so as to connect all the units, care settings, and organizations involved into DF care. Gray proposes the Population Medicine approach [53] as a means of encouraging clinicians to focus on the entire pathway and not only on the clinical phases they are in charge of. Clinicians are asked to share responsibility ‘*to the population they serve, to the patients they never see, as well as to the patients who have consulted or been referred’* [49, p. 200] as “public health professionals”. This approach stops clinicians being responsible for a specific phase or ward, and instead makes them jointly responsible for the network of services, the outcomes and the resources linked to a specific pathway.

## Methods

In order to diffuse Integrated Care into DF care, three main strategies were adopted:

– Using the MeS-Lab PES to encourage clinicians to focus on geographic variation;
– Using quantitative and qualitative information at both the HA and patient levels to inform clinicians’ debate and to spread the Population Medicine perspective;
– Creating a stable community of professional practice in order to discuss data, identify best practices of Integrated Care and share improvement actions [31,32].

The project started in 2012 and was carried out over a period of about two years.

### Encouraging clinicians to focus on geographic variation

Firstly, the diabetes-related lower limb major amputation rate (DRMAR) was included in the MeS-Lab PES (see Supplementary File 1 for a definition of DRMAR).

The most important elements of the MeS-Lab PES were then applied to the DRMAR. In fact, MeS-Lab PES proved to be effective in facilitating the comprehensiveness of the performance information and in supporting improvement [33,34,35,54]. These elements include:

– The use of five coloured assessment bands based on the benchmark results: red - poorest performers; orange - poor; yellow - average; green - good; and dark green - best. Benchmarking enables performance to be evaluated by assigning the five bands considering the overall average and the distribution of HA results;
– The inclusion of the DRMAR in the dartboard diagram representing the overall performance of each HA. The dartboard has five bands: when performance is excellent, the results are positioned from the central dark green zone for best performers towards the outer strips, with red representing the poorest performance (Supplementary File 2 includes an example of the dartboard).
– The public disclosure of results on a website, http://www.performance.sssup.it/toscana which not only provides stakeholders with all the information available but also through benchmarking creates a “competition” between clinicians based on reputation.
– The setting of quantitative targets: the Regional Government sets a quantitative target for each HA for each indicator included in the MeS-Lab PES.
– A link between the target achievement and the 20% variable share of HA CEOs’ annual salaries.

### Using quantitative and qualitative analyses to spread the Population Medicine approach

In order to spread the Population Medicine approach, we provided clinicians with specific analyses that mixed different issues (service utilization, outcomes, costs), different levels of analysis (HAs and individual patients), and different methods and sources (administrative data analyses and patient surveys).

In addition, population-based data was cross-checked based on Small Area Variation Analyses (SAVAs) and data were calculated at the delivering facility level.

By benchmarking the utilization rates of healthcare services (e.g. surgical intervention, diagnostic procedures or hospital admission rates), the SAVA compared the costs and the outcomes, between geographic areas and comparable populations [55]. The delivery-perspective provided information regarding which facilities delivered specific services (e.g. performed surgical interventions) or directly generated costs.

Cross-checking these analyses provided a preliminary mapping of the real patient pathways, which usually involved several clinicians, wards and facilities in different HAs. This process challenged the inward-looking perspective and the silo-working approach of clinicians who focused only on patients seen in their own facilities or on the specific phase they managed. Regular benchmarking was essential in guiding data interpretation and discussions between clinicians regarding the potential lack of Integrated Care.

All the analyses on service utilization, outcomes and costs were provided not only for each HA but also at a patient level.

The first level provided data on volumes and estimated expenditure for the services delivered by each HA for the various LHA populations. These analyses checked which services were globally delivered and the overall impact on LHA budgets.

Patient pathways were tracked across different services and facilities in order to help clinicians understand whether appropriate care was being delivered to each patient. These analyses focused on a cohort of 190 diabetic residents in Tuscany who had been amputated in 2011. The database was created with a record linkage of administrative flows between 2009 and 2012 regarding hospitalizations, outpatient visits, diagnostic tests, and drug consumption. Each patient’s clinical history considered the one year prior to and after hospitalization for major amputation.

Finally, the analyses discussed by clinicians was based mainly on administrative data, but also on a patient survey of specific phases of DF care.

### Engaging professionals

From the very beginning, the program involved the DF clinicians in discussing the analyses, identifying barriers to integration and best practices and proposing improvements. The process of engaging clinicians was based on the principles of action research [31,32,56,57,58] and involved mapping the organization of DF pathways in each HA and organizing meetings to discuss data and practices.

Firstly, the two MeS-Lab researchers involved in the action research project and a representative of the Regional Commission for Diabetes designed a questionnaire to uniformly map the DF pathways (the Regional Commission for Diabetes is a technical consulting body for the Regional Administration made up of clinicians and technicians working in the regional healthcare system specialized in diabetes care). The questionnaires analyzed eight areas where integration was considered essential: screening, admissions and visits, revascularization procedures, surgery, urgent pathways, follow-up and continuity of care, education for patients and caregivers, training clinicians, and information systems.

Researchers then visited the Diabetic Foot Outpatient Clinics in the 12 LHAs and in the three THs and mapped the organization of the DF pathways through the questionnaires in collaboration with the team leaders of the units. These clinicians were involved because of their role in coordinating the services and professionals involved in DF care. In addition, visits were planned so as to create a trusting relationship between the researchers and clinicians, in order to openly discuss their practices.

Researchers then arranged the first plenary meeting with clinicians and their DF-teams (e.g. diabetologists, nurses, podiatrists), the managers of the Health Departments of each HA, the representatives of the Regional Government, GPs and the Tuscany Diabetic Patient Association, with a total of 47 people.

The plenary meetings between researchers, managers and clinicians were then conducted around every three months to discuss the analyses and the results of the mapping, to identify good performance and Integrated Care best practices as well as to propose improvement strategies. Clinicians often suggested the calculation criteria of the quantitative analyses [31,32].

## Results

The combined implementation of these strategies enabled those working on the project to foster Integrated Care in their local context. The project:

- Shifted the focus to the reduction of geographic variations by using the MeS-Lab PES;
- Made clinicians more accountable for the outcomes of their local populations and enabled them to foster collaboration with other professionals in their local communities as required by the Population Medicine approach;
- Enabled clinicians to identify the gaps in integration and the organizational best practices and improve Integrated Care by tackling weaknesses.

In this section, the results are presented considering the main output of the three strategies. In addition, the specific case study of the Teaching Hospital in Pisa is described i.e. the Health Authority that faced major problems in terms of Integrated Care.

### Focusing on geographic variation

**Figure 2:**
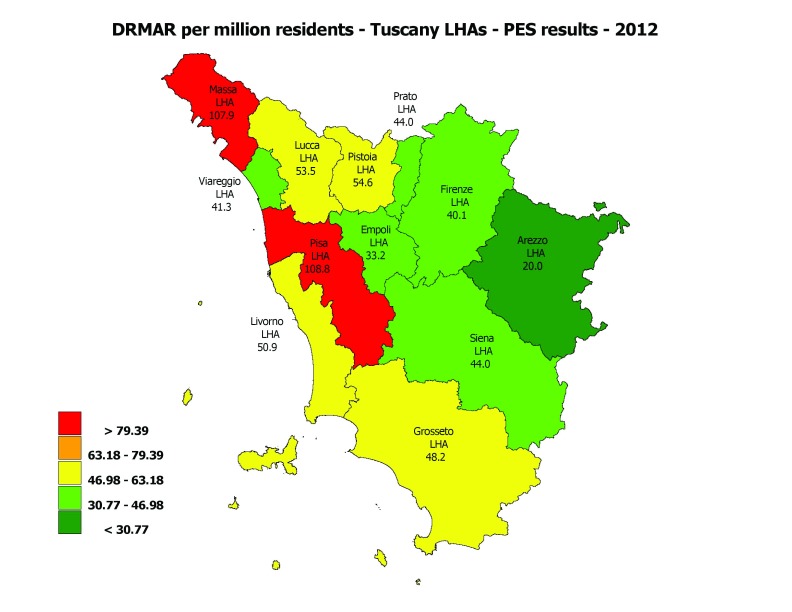
Diabetes-related lower limb major amputation rate per million residents – MeS-Lab Tuscany PES results – 2012. Source: MeS-Lab.

The first step was to shift the clinicians’ focus on the variation in the DF care, by combining all the PES elements considering the most DF outcome: the DRMAR. The indicator was highlighted in a geographical map with coloured bands based on benchmarking (Figure 2). Data were also published on the Tuscany PES website.

**Figure 3:**
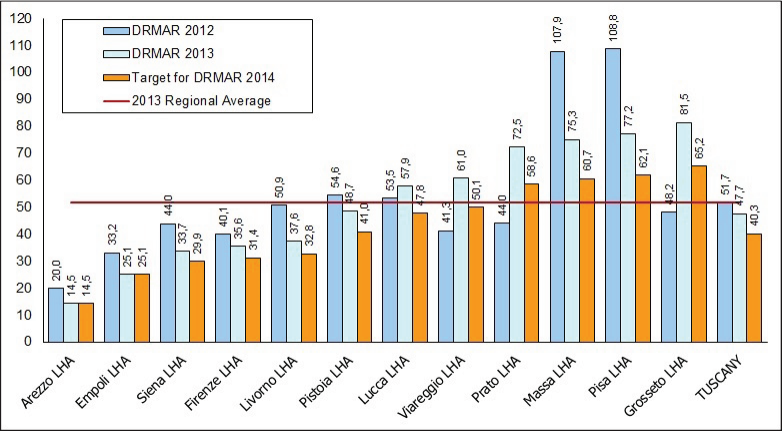
LHA DRMAR targets in Tuscany for 2014. Source: MeS-Lab.

In addition, the Regional Government set a specific quantitative target for each LHA and requested that the poorer performers should strive to reach the same levels as the best performers (Figure 3).

### Spreading the Population Medicine Approach

**Figure 4:**
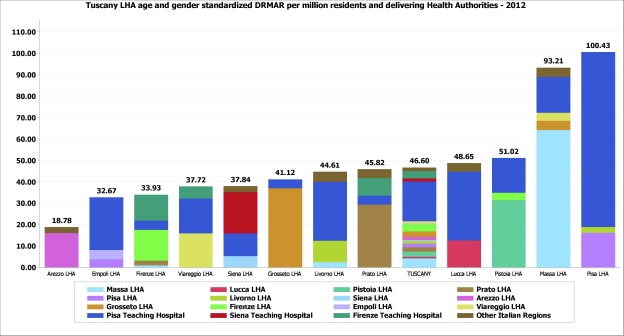
Age and gender standardized diabetes-related lower limb major amputation rate per million residents in Tuscany, 2012. Details for the delivering Health Authority. Source MeS-Lab.

Age and gender risk-adjustments were added to the DRMAR, showing the persistence of variations even after controlling for the main population needs (Figure 4) and highlighting how each provider contributed to the overall rate.

Crosschecking the population and delivery levels showed the clinicians of the Teaching Hospital of Pisa how the amputations delivered in their facilities were contributing to the poor performance of the LHA in their geographical area (Pisa-LHA). In fact, in 2012, the risk of being amputated in the area of Pisa-LHA was fivefold higher than the risk in the Arezzo-LHA. Thus, the THs and the LHAs of Pisa, Firenze and Siena were assigned a common quantitative target to reduce the DRMAR in their contexts in order to share the joint responsibility of their populations.

For the 190 amputated patients included in the DF cohort, the researchers and clinicians examined whether or not each patient had received expected and appropriate care: two patients amputated in 2011 did not receive any outpatient visits and were not hospitalized within a year prior to their amputation. These cases showed that there were problems with Primary Care and Diabetic Foot Outpatient Clinics in fostering prevention, early diagnoses and early treatments. The audit process for these 190 patients showed how enhancing Integrated Care among providers was necessary, notwithstanding the overall good results of Tuscany compared to other regions in Italy.

**Figure 5:**
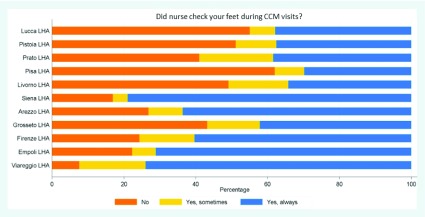
Diabetic patient experience regarding foot check-ups in Tuscan LHAs – Survey 2012. Source: MeS-Lab.

To highlight the lack of coordination with Primary Care, the MeS-Lab provided clinicians with a survey to assess the perceived quality of the Tuscan Program in the Chronic Care Model [59,60]. Patients declared that the foot check-ups during Primary Care visits were the weakest point provided by GPs and their staff. In fact, foot check-ups showed an overall lower compliance with respect to other diabetes check-ups (weight, glycaemia, etc.) and they were also not performed uniformly (Figure 5) (See the Supplementary File 3 for the complete survey method).

The population-based perspective used for the cost-analyses enabled clinicians to discuss how the services provided in the DF pathways impacted on LHA resources. The audits on resources for DF pathways were carried out by dividing the overall healthcare expenditure for the 190 patients in the cohort (approximately € 6 million) into several components (e.g. hospitalizations, outpatient visits, diagnostic and laboratory tests, and drugs delivered within one year before and one year after major amputations).

**Figure 6:**
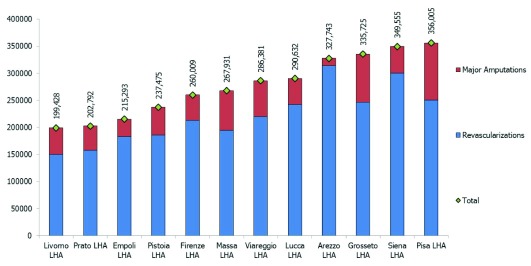
Estimated LHA expenditure (DRG) of hospitalizations for diabetes-related revascularizations and lower limb major amputations per 100.000 residents – Average of the four-year period between 2009 and 2012. Source: MeS-Lab.

The researchers calculated the estimated LHA expenditure (based on DRG-tariffs) of hospitalizations for diabetes-related revascularization of lower limbs by considering the average expenditure between 2009 and 2012. The expenditure estimate for DRMAs was collected with the same criteria. In order to compare the impact of these two items on LHA budgets, the estimated expenditure was then re-proportioned per 100,000 residents (Figure 6) (see table in Supplementary File 4).

Thus, DRMAR hospitalization expenditure could be considered as an opportunity cost for delivering other services for preventing major surgical interventions (e.g. revascularizations). Figure 6 highlights that LHA expenditures varied sharply considering the overall values and the potential reallocation from expenditure for amputations to expenditure for preventive treatments. The best performer (the Arezzo-LHA) showed a strongly oriented mix towards revascularizations and did not account for the lower overall costs. The clinicians thus did discuss a more relevant topic for their daily practice, rather than the “traditional” issue of pure savings: the potential reallocation towards services with greater value for money, which can be achieved by enhancing coordination between providers and shifting from a provider-centred to a population-based and patient-centred perspective.

The population-based approach helped the clinicians to shift their focus from the costs of the productive factors they directly managed in their wards (personnel costs, etc.) to the impact of the overall expenditure for DF patients on their own LHA overall budgets. The new accountability also included THs because of their role in delivering DF care for the residents in their LHA.

### Engaging professionals: shared solutions for implementing Integrated Care

During the mapping phase and the periodic meetings, the community of practice outlined the barriers to Integrated Care in each local context and identified the best organizational practices.

Table 1 summarizes the mapping results divided into eight areas in the questionnaire. The results highlighted what issues were hindering Integrated Care practice and where.

**Table 1:** Summary of the organizational mapping.

Area	Mapping results

Screening, admissions and visits	Professionals pointed out coordination problems with Primary Care (PC) regarding the prompt identification and management of ulcers/complications, leading to late diagnoses. Patients were therefore often admitted to the outpatients clinics with severe conditions and without previous DF assesment by their GPs. This was confirmed by the survey presented in Figure N.5.: foot check-ups by PC professionals were not a comman practice. Outpatient clinics also differed in the scheduling procedures for visits and diagnostics exams. Moreover, some HAs did not schedule dedicated slots in their radiology departments in agreement with the diabetology departments.
Revascularization procedures	The organization of this phase greatly differed between HAs. Some LHAs did not have catherization labs to perform lower-limb revascularization procedures so they had to refer their patients to other HAs. This implied a greater need for coordination among these providers. Other HAs provided some schedule hours during the week for the revascularization of lower limbs. In some HAs, revascularizations were usually performed without involving the diabetologists.
Surgery	In some HAs, interventions and follow-up treatments were often planned without involving the diabetologists. Lack of coordination occured both before and after surgery. Only in some HAs, diabetologists directly performed basic and minor surgical procedures.
Urgent path	Some professionals identified barriers to flexible and “fast track”, access, exams, revascularization procedures and interventions for patients with urgent needs because of a lack of coordination with other professionals.
Follw-up and continuity of care	Communication with PC professionals was also considered a critical aspect after discharges with regards to the management of lesions, wounds, ulcers and specific medications.
Education for patients and caregivers	Only in some organizations, educations for the prevention and treament of ulcers and lesions was provided for both individuals and groups of patients and caregivers. PC professionals were often not well-trained in preverting and recognizing DF problems and providing appropriate education for patients and caregivers.
Training professionals	Perodic educational meeting on prevention, treatment and team building, especially between diabetologists and PC professionals, were not held in every organization. These meetings should be held more in large hospitals (such as THs) and in those areas where the care path is fragmented among LHAs and THs.
Information Systems	The development and implementation of information systems are very different in each organization. Only some HAs quickly collected comprehensive information about their patients over time and easily shared them with other departments.

The LHA of Arezzo, which was the national and regional best performer in terms of outcomes, was identified as the organization combining the best organizational practices.

The Arezzo DF-team identified clear steps for DF services in collaboration with the clinicians in other wards and settings. For instance, daily contacts between diabetologists and other clinicians led to the scheduling of weekly slots for diagnostic tests and revascularizations based on the analysis of demand. They also ensured fast-track pathways for urgent cases. In addition, communication was enhanced between wards: all the clinicians were fully aware of the DF issue and promptly informed the DF-team in case of inpatient DF complications, thus reducing both bottlenecks and late diagnoses/treatments. The cardiology department provided equipment for the DF-team in their ward so that the DF-team could directly care for hospitalized DF patients, regardless of organizational boundaries. In addition, the DFOC information system was completely integrated with the other systems implemented in other wards. Finally, the DFOC clinical staff performed minor surgical procedures thus avoiding the need for surgeons for basic treatments, and therefore reducing bottlenecks. Integration with healthcare services outside Arezzo’s hospital was also established: GPs had clear pathways for both DFOC activities and diagnostic exams through simple slot scheduling schemes. In addition, the DF-team participated in all the training initiatives organized at the Primary Care level in order to enhance awareness of DF complications.

After summarizing the issues that arose from the mapping phase and taking into account the case of Arezzo, the clinicians and researchers identified three main sources of barriers to Integrated Care, linked to the categories identified in the background section in terms of integration between:

1. units within the same organization, the main barrier was a lack of coordination among clinicians within an organization (e.g. a hospital). General surgeons, orthopedics, cardiologists, vascular surgeons, interventional radiologists did not always coordinate with the DF-team (diabetologists, podiatrists). Instead, the DF-team should be quickly identifiable and involved in the decisions concerning the pathway regardless of organizational boundaries.
2. different care settings, the main barrier was the lack of coordination between hospital, community and primary care. Clinicians experienced a lack of information flow between care settings, such as between outpatient clinics and general practitioners. This also included a lack of mutual training between clinicians on essential topics for DF care and prevention.
3. different institutions, the main barrier was the lack of coordination between HAs. HAs in the same geographical area or in neighboring areas experienced stronger barriers to Integrated Care because of their “artificial” organizational boundaries. This occurred especially when a TH was the hospital care provider for another authority, the LHA, the other healthcare services (e.g. primary and community care).

The engagement approach used to support the mapping and the discussions was needed to compare practices and data. In fact, HA representatives participated in every meeting and frequently asked for additional in-depth analyses of their local contexts. The results of this engagement process enabled them to share organizational best practices and clinicians were encouraged to propose solutions for the issues identified. This occurred both at the regional level, through the shared proposal for redesigning DF care organization, and at the local level, where each DF-team proposed initiatives to tackle the issues in their own context. In particular, the case of Pisa TH is an example of how the project changed the attitude of clinicians towards Integrated Care.

Finally, the engagement process succeeded in convincing clinicians to apply the same approach on other diabetes-related pathways (hypoglycemia events, etc).

#### The diffusion of Integrated DF Care at the regional level

A proposal to re-design the DF pathway in Tuscany towards greater Integrated Care was sent to the Regional Commission for Diabetes. The document was based on the results of the project and provided organizational recommendations for tackling the three barriers. The document was approved by the Regional Health Council in July 2013, aimed at updating the future the Regional Administration Act 1304 dated 9/12/2003 on the organization of the DF care.

The document focused on the following items, and was particularly inspired by the results achieved in Arezzo:

- role of the diabetologist as the coordinator of the entire care with a pathway-oriented approach, regardless of organizational boundaries;
- implementation of flexible and shared fast-track pathways for urgent treatments and diagnostic tests;
- interdisciplinary collaboration of clinicians involved in the pathway at the hospital level (radiologists, podiatrists, diabetologists, vascular and orthopedic surgeons, cardiologists, etc.);
- training for diabetologists in basic surgical skills;
- clear and periodic communication, coordination and training initiatives between hospitals and primary care professionals in order to improve both preventive and follow-up care;
- reinforcement of the network of professionals in the different HAs, not just between the diabetologists but also between GPs, nurses and podiatrists.

#### The case of the Teaching Hospital in Pisa

Pisa-LHA is one of 12 Tuscan Local Health Authorities serving a population of about 340,000 residents. Hospital care in the city of Pisa is delivered by the Pisa Teaching Hospital (Pisa-TH) and not directly by the LHA, thus leading to complex coordination between hospital, community and primary care services.

The results of the Pisa-LHA DRMAR were expected to be good, considering that Pisa-TH was the regional referral center for diabetes and it was the only hospital in the Region of Tuscany with a specific department managed by diabetologists. Conversely, during the three-year period 2010-2012, Pisa-LHA had one of the highest regional values.

The analyses carried out during the project identified the Pisa area as experiencing all three barriers to Integrated Care:

– integration between units within the organization: there were problems in internal coordination due to the high number of clinicians and wards involved in caring for DF patients;
– integration between care settings and institutions: there was a lack of coordination between care settings and Health Authorities. These Pisa-TH coordination issues were amplified because of the organizational boundaries with the Local Health Authority. Indeed, the division between the independent Pisa-TH in charge of hospital care, the Pisa-LHA in charge of community and home services, and the self-employed General Practitioners rewarded on a capitation-basis sharply increased lack of coordination. All the analyses carried out during the project confirmed these problems.
– For instance, the Pisa area:
– accounted for the lowest adherence to the screening for foot complications in Primary Care, as previously shown in Figure 5;
– had the greatest need for reallocating resources from amputations towards preventative care, as shown in Figure 6;
– accounted for the highest DRMAR in Tuscany due to the high number of amputations affecting the residents in Pisa-LHA and delivered by the Pisa-TH.

Initially, the role of the Pisa-TH as DF regional referral center was seen as a reason for not questioning the Pisa-TH about the persistently and increasingly high DRMAR of the Pisa-LHA. However, the project was effective in shifting the focus from a delivery- to a population-based perspective. Indeed, the DFOC of the Pisa-TH started to re-consider its role as the most important delivering facility for the inhabitants of Pisa and therefore accountable for the results of the Pisa-LHA, regardless of the organizational boundaries between the authorities.

Thus, the DFOC-team began a constructive improvement process to deal with the three areas lacking integration.

Firstly, concerning integration between units within the organization, various improvements were carried out in order to foster structured relations with the other clinicians involved in the DF care in the Pisa-TH. MeS-Lab researchers provided more detailed information on the pathways of Pisa-LHA residents amputated in the Pisa-TH (length of stay, operation ward, discharge ward, age, gender, educational level, previous screening and revascularization, etc.). The DF-team then carried out a comprehensive analysis of the DF care provided in the Pisa-TH. Before the project, the focus of the DFOC clinicians was only on the amputations they had performed or on those performed by other Pisa-TH clinicians for patients previously cared for in the DFOC. Hence, the patient pathways for amputations in the Pisa-TH without DFOC involvement (48%) were not analysed and discussed. Moreover, this lack of involvement of the diabetologists resulted in a less conservative surgical approach being provided in the other departments (in case of unavoidable amputations due to the severe health status of the patients, it is preferable to provide a conservative surgical approach, e.g. below the ankle, because it enables patients to walk using special shoes).

The mapping phase showed that the PISA-TH pathway for DF patients cared for by the DFOC was well staffed and organized. The DFOC included a team of podiatrists with resources and beds directly managed by the Diabetology Department. In addition, the DF-team performed minor and conservative amputations directly, with scheduled slots in the surgical rooms and in the cath-lab for revascularizations.

However, in the complex organization of the Pisa-TH, some patients left or did not start this path. The lack of shared decisions and a defined pathway was because in the Pisa-TH there were several practitioners in both clinician-led and academic-led wards, thus multiplying the interactions between professionals and departments and increasing the chances of poor coordination and miscommunication. This confirmed the need for greater integration among the several Pisa-TH clinicians and services, and it raised concerns about equitable treatment for DF patients.

Consequently, with the detailed analyses of patient pathways, the DF-team carried out internal audits with the other clinicians involved in DF care to map the internal pathway of each amputated patient; in particular, those who had not been previously cared for by the DF-team and had been hospitalized for amputations by the emergency department.

The results were presented to the vascular surgeons and a multi-disciplinary audit was then carried out to share whether and when to perform surgical procedures. These results were also discussed by the DF community of practice in the plenary meetings.

With regard to the integration between care settings, the key focus was to reduce the lack of coordination with Primary Care. The DFOC director decided to organize training courses for Pisa-LHA community nurses in charge of the DF screening phase. The initiative to overcome the TH boundaries and to interact with Primary Care structures of the Pisa-LHA area (in some cases, over one-hour distance by car from Pisa-TH) confirmed the strong commitment to the Population Medicine approach.

With regard to the integration between institutions, Pisa-TH fostered the overall coordination with the Pisa-LHA. The internal audits carried out by Pisa-TH clinicians were also adopted externally in order to map all the patient pathways that were shared by both institutions (i.e. the Pisa-TH and the other peripheral hospitals managed by the LHA). Periodical meetings involving health clinicians and managers of the two institutions were thus organized.

All these steps enhanced stronger collaboration and joint accountability by the Pisa-LHA and the Pisa-TH for the DRMAR results, thus overcoming organizational boundaries.

Finally, initial evidence of outcome improvements was found in the DRMAR indicator: after the start of the project, the persistent increase in the Pisa-LHA DRMAR stopped and in 2014 the Pisa-LHA DRMAR registered an overall decrease of 38% with respect to 2012.

## Discussion

Based on the study presented in this paper, some complementary steps should be carried out to tackle the barriers to integration and to drive consistent improvement in those pathways where Integrated Care is particularly related to outcomes [61].

This approach includes: the use of a transparent and systematic PES along with stimuli for driving focus on geographic variation; the diffusion of a Population Medicine approach to shift professionals from a silo-working to Integrated Care practice; and the engagement of professionals as the key to promoting concrete improvement.

The implementation of a Performance Evaluation System comparing benchmarking data and the use of effective tools to represent performance (dartboard and geographic maps) are the first steps in order to be able to highlight geographic variations and best practices. Public disclosure of these data raises professionals’ awareness, leading to a “reputational competition” [25,30,62,63]. However, the public comparison of results and “naming and shaming” are not enough to ensure change [25,30]. In fact, to spread Integrated Care and to improve outcomes professionals need to increase their awareness of any lack of integration within the system. Measuring and evaluating should thus be complemented with in-depth analyses to audit practices. This should be carried out through quantitative analyses based on administrative data regarding patient pathways and through “narrative” tools based on real patient stories. This evidence should inform audits in order to recognize and investigate sentinel events [64] when Integrated Care fails in daily practice. These analyses should thus go beyond the single provider level and should cross-check the delivery- with the population-based perspective.

In addition, discussing analyses with clinicians comparing service utilization and outcomes with costs shows that, in most cases, it is possible to achieve greater quality of care and better outcomes without increasing costs. Indeed, financial sustainability can be achieved through resource reallocation rather than through across-the-board budget cuts. As in the case of the DF, fostering this issue in those multi-provider and multidisciplinary pathways where Integrated Care is strongly connected with quality of care, can foster value for money strategies and should become a financial priority.

To implement improvement actions towards Integrated Care an engagement strategy [65,66] should be pursued. Based on our experience, this can be done firstly by mapping organizational practices in each Health Authority and, secondly, by involving all the professionals in periodic peer-review meetings. Mapping takes into account the specificity of each local context and thus enables professionals to outline their own experience and environment. This is the first step for professionals to be engaged in a permanent community of practice. In this environment, clinicians can systematically discuss data and experience and receive constructive feedback through peer-pressure [67].

This approach actually enabled professionals to outline best practical models of Integrated Care and share improvement solutions. Hence, the community of practice allowed also to identify and reward the best performers through peer-reputation.

Finally, the community of practices needs to share a common set of values that enables professionals to feel jointly accountable for the entire pathway and not only for the phase they are in charge of. In this respect, the Population Medicine approach embodies this key message and could be identified as the set of work values that should shape the professional culture in those organizations seeking more Integrated Care.

The approach presented in this paper was framed specifically in Tuscany, and in a specific highly-specialized pathway, the DF care. However, our approach could be applied to all areas and to all care pathways where Integrated Care may be the leading factor to improve outcomes.

In fact, for all those health services whose results are strongly linked to Integrated Care, a new specific category regarding the determinants of geographic variation could be identified. Wennberg and colleagues [17,18,19], in studying the determinants of geographic variation, suggested three main categories: effective care, supply-sensitive care, and preference-sensitive care [17,18,19,20,21,22,23,24,68]. In the first category, the authors consider individual procedures where clinical evidence is available and variation should be reduced.

Based on our experience, ‘effective care’ should be split into two subcategories (Table 2). In fact, some care paths cannot be evaluated individually by considering the individual procedures and treatments but need to be considered with a pathway perspective, where Integrated Care is the significant factor that affects outcomes along the entire care continuum.

**Table 2:** The determinants of geographic variation [adapted from 17,18,19,20,21,22,23,24,68].

Categories of variation in medical care		Actions

Effective Care of an individual service or procedure (e.g. minimal volumes for specific surgical precedures to ensure patients’ safety and better outcomes)	Refers to services of proven values and without significant tradeoffs: the benefits of these services outweigh the risks	Reduction
Effective care of an Integrated Care pathway	Refers to services whose variation is due to a lack of integration throught the entire care pathway	Reduction
Supply-sensitive care	Represents service for which human and the availability of technical resources (e.g. physicians, hospital beds) strongly influence the amount of care delivered	Reduction
Preference-sensitive care	Comprises care for conditions that have more than one treatment option, each with its own benefits and tradeoffs	Follow patient preferences

Therefore, as in the case presented in this paper, it is possible to recognize services whose geographic variation is related to a lack of Integrated Care. For these services, Integrated Care and unwarranted geographic variation are connected and a comprehensive strategy to reduce equity gaps by diffusing integration should be carried out.

## Conclusion

In a Beveridge Healthcare System, which pursues universal coverage and equity, clinicians should be engaged in a cultural change where their work is less constrained by organizational boundaries. Clinicians should be steered towards the creation of overall value for patients in a population-based perspective and the adoption of Integrated Care as a systemic approach throughout the entire pathway.

Services whose outcomes are particularly related to Integrated Care should be fully analysed by a stable community of practitioners in order to identify and tackle barriers to integration. This will create a healthcare system where clinicians share joint accountability for both the outcomes and the costs of the care pathways in which they are involved and not just for the patients they directly care for, the phases for which they are in charge of, or the productive factors they manage. This process enables healthcare professionals and managers to share their common commitment towards the principle of equity pursed by Beveridge healthcare systems.

## Acronyms

DF: Diabetic Foot

PES: Performance Evaluation System

MeS-Lab: Management and Health Laboratory

LHA: Local Health Authority

HA: Health Authority

DRMAR: Diabetes-related lower limb major Amputation rate

DRG: Diagnostic-Related Group

DFOC: Diabetic Foot Outpatient Clinic

TH: Teaching Hospitals

CCM: Chronic Care Model

PC: Primary Care

## Supplementary Files

The supplementary files for this article can be found as follows:

- **Supplementary File 1:** Appendix. ijic-16-2-1991-s1.pdf

## References

[1] Reed J, Cook G, Childs S, McCormack B (2005). A literature review to explore integrated care for older people. International Journal of Integrated Care.

[2] Vondeling H (2004). Economic evaluation of integrated care: an introduction. International Journal of Integrated Care.

[3] Kodner D (2009). All together now: a conceptual exploration of Integrated Care. Healthcare Quarterly.

[4] Gröne O, Garcia-Barbero M (2001). Integrated care: A position paper of the WHO European office for integrated health care services. International Journal of Integrated Care.

[5] National Institute for Health Care Excellence (2015). Internal Clinical Guidelines team – Diabetic foot problems – Prevention and management. NICE Clinical Guideline. Methods, evidence and recommendations.

[6] Armstrong DG, Bharara M, White M, Lepow B, Bhatnagar S, Fisher T (2012). The impact and outcomes of establishing an integrated interdisciplinary surgical team to care for the diabetic foot. Diabetes/Metabolism Research and Reviews.

[7] El Sakka K, Fassiadis N, Gambhir RPS, Halawa M, Zayed H, Doxford M (2006). An integrated care pathway to save the critically ischaemic diabetic foot. International Journal of Clinical Practice.

[8] Donohoe ME, Fletton JA, Hook A, Powell R, Robinson I, Stead JW (2000). Improving foot care for people with diabetes mellitus – a randomized controlled trial of an integrated care approach. Diabetic Medicine.

[9] World Health Organization (2005). World Diabetes Day: too many people are losing lower limbs. http://www.who.int/mediacentre/news/releases/2005/pr61/en/.

[10] Wrobel JS, Charns MP, Diehr P, Robbins JM, Reiber GE, Bonacker KM (2003). The relationship between provider coordination and diabetes-related foot outcomes. Diabetes Care.

[11] Canavan R, Unwin NC, Kelly WF, Connolly VM (2008). Diabetes- and Non-diabetes-Related Lower Extremity Amputation Incidence Before and After the Introduction of Better Organized Diabetes Foot Care. Diabetes Care.

[12] Krishnan S, Nash F, Baker N, Fowler D, Rayman G (2008). Reduction in Diabetic Amputations Over 11 years in a Defined U.K. Population. Diabetes Care.

[13] Doggen K, Van Acker K, Beele H, Dumont I, Félix P, Lauwers P (2014). Implementation of a quality improvement initiative in Belgian diabetic foot clinics: feasibility and initial results. Diabetes/Metabolism Research and Reviews.

[14] Grant J (2010). What does it take to make integrated care work?. http://www.mckinsey.com/industries/healthcare-systems-and-services/our-insights/what-does-it-take-to-make-integrated-care-work.

[15] Goodwin N (2013). How do you build programmes of integrated care? The need to broaden our conceptual and empirical understanding. International Journal of Integrated Care.

[16] Goodwin N (2013). Taking integrated care forward: the need for shared values. International Journal of Integrated Care.

[17] Wennberg JE, Gittelsohn A (1973). Small area variations in health care delivery; a population-based health information system can guide planning and regulatory decision making. Science.

[18] Wennberg JE (1999). Understanding geographic variations in health care delivery. The New England Journal of Medicine.

[19] Wennberg JE, Fisher ES, Skinner JS (2002). Geography and the debate over Medicare reform. Health Affairs.

[20] Mulley AJ (2010). Improving productivity in the NHS. British Medical Journal.

[21] Appleby J, Raleigh V, Frosini F, Bevan G, Gao H, Lyscom T (2011). Variations in Healthcare. The good, the bad and the inexplicable.

[22] NHS Right Care (2011). The NHS Atlas of Variation in Healthcare. Reducing unwarranted variation to increase value and improve quality.

[23] OECD (2014). Geographic Variations in Health Care: What do we know and what can be done to improve Health System Performance? OECD Health Policy Studies 2014.

[24] Nuti S, Seghieri C (2014). Is variation management included in regional healthcare governance systems? Some proposals from Italy. Health Policy.

[25] Leatherman S, McCarty D (1999). Public disclosure of health care performance reports: Experience, evidence and issue for policy. International Journal for Quality in Healthcare.

[26] Marshall MN, Shekelle PG, Leatherman S, Brook H (2000). Public disclosure of performance data: Learning from the US experience. Quality in Health Care.

[27] Hibbard JH, Stockard J, Martin T (2003). Does publicizing hospital performance stimulate quality improvement efforts?. Health Affairs.

[28] Shekell PG, Lim YW, Mattke S, Damberg C (2008). Does public release of performance results improve quality of care: a systematic review.

[29] Fung CH, Lim Y, Mattke S, Damberg C, Shekelle PG (2008). Systematic review: the evidence that publishing patient care performance data improves quality of care. Annals of Internal Medicine.

[30] Bevan G, Wilson D (2013). Does ‘naming and shaming’ work for schools and hospitals? Lessons from natural experiments following devolution in England and Wales. Public Money & Management.

[31] Passmore W, Reason P, Bradbury H (2001). Action research in the workplace: the socio-technical perspective. Handbook of Action Research.

[32] Schein EH, Reason P, Bradbury H (2001). Clinical inquiry/research. Handbook of Action Research.

[33] Nuti S, Seghieri C, Vainieri M (2012). Assessing the effectiveness of a performance evaluation system in the public healthcare sector: some novel evidence from the Tuscany Region experience. Journal of Management and Governance.

[34] Nuti S, Seghieri C, Vainieri M, Zett S (2012). Assessment and improvement of the Italian Healthcare system: first evidences from a pilot national performance evaluation system. Journal of Healthcare Management.

[35] Nuti S, Vola F, Bonini A, Vainieri M (2016). Making governance work in the health care sector: evidence from a ‘natural experiment’ in Italy. Health Economics, Policy and Law.

[36] Agenzia Nazionale per i servizi sanitari regionali (Age.na.s) Programma Nazionale Esiti (National Agency for Regional Health Services. National Outcomes Evaluation Programme). http://95.110.213.190/PNEed15/index.php.

[37] Bakker K, Apelqvist J, Schaper NC (2012). Practical guidelines on the management and prevention of the diabetic foot 2011. Diabetes/Metabolism Research and Reviews.

[38] American Diabetes Association (2008). Economic costs of diabetes in the U.S. in 2007. Diabetes Care.

[39] Driver VR, Fabbi M, Lavery LA, Gibbons G (2010). The costs of diabetic foot: the economic case for the limb salvage team. Journal of Vascular Surgery.

[40] Mainz J (2003). Defining and classifying clinical indicators for quality improvement. International Journal for Quality in Health Care.

[41] Schofield CJ, Libby G, Brennan GM, MacAlpine RR, Morris AD, Leese GP (2006). Mortality and hospitalization in patients after amputation: a comparison between patients with and without diabetes. Diabetes Care.

[42] Kodner DL, Spreeuwenberg C (2002). Integrated care: meaning, logic, applications, and implications – a discussion paper. International Journal of Integrated Care.

[43] Øvretveit J (1998). Integrated Care: Models and Issues. Briefing Paper.

[44] Gröne O, Garcia-Barbero M (2002). Trends in Integrated Care – Reflections on Conceptual Issues.

[45] Schrijvers G, Goodwin N (2010). Looking back whilst moving forward: observations on the science and application of integrated care over the past 10 years and predictions for what the next 10 years may hold. International Journal of Integrated Care.

[46] Valentijn PP, Schepman SM, Opheij W, Bruijnzeels MA (2013). Understanding integrated care: a comprehensive framework based on the integrative functions of primary care. International Journal of Integrated Care.

[47] Nolte E, Pitchforth E (2014). What is the evidence on the economic impacts of integrated care? Policy Summary 11.

[48] Shaw S, Rosen R, Rumbold B (2011). What is Integrated Care? Research Report.

[49] Lewis R, Rosen R, Goodwin N, Dixon J (2010). Where Next for Integrated Care Organisations in the English NHS?.

[50] Leutz W (1999). Five laws for integrating medical and social services: lessons from the United States and the United Kingdom’. The Millbank Quarterly.

[51] Nies H, Berman PC (2004). Integrating Services for Older People: A resource book for managers.

[52] Shortell SM, Gillies RR, Anderson DA (1994). The new world of managed care: creating organized delivery systems. Health Affairs.

[53] Gray JAM (2013). The shift to personalised and population medicine. Lancet.

[54] Pinnarelli L, Nuti S, Sorge C, Davoli M, Fusco D, Agabiti N (2012). What drives hospital performance? The impact of comparative outcome evaluation of patients admitted for hip fracture in two Italian regions. BMJ Quality & Safety.

[55] Diehr P (2005). Small Area Variation Analysis. Encyclopedia of Biostatistics.

[56] Whyte WE (1991). Participatory action research.

[57] Bradley EH, Curry LA, Ramanadhan S, Rowe L, Nembhard IM, Krumholz HM (2009). Research in action: using positive deviance to improve quality of healthcare. Implementation Science.

[58] Bardach E (2012). A practical guide for policy analysis. The eightfold path to more effective problem solving.

[59] Wagner EH (1998). Chronic disease management: What will it take to improve care for chronic illness?. Effective Clinical Practice.

[60] Barr VJ, Robinson S, Marin-Link B, Underhill L, Dotts A, Ravensdale D (2003). The expanded chronic care model: An integration of concepts and strategies from Population Health Promotion and the Chronic Care Model. Healthcare Quarterly.

[61] Maruthappu M, Hasan A, Zeltner T (2015). Enablers and barriers in implementing integrated care. Health Systems & Reform.

[62] Ettorchi-Tardy A, Levif M, Michel P (2012). Benchmarking: A Method for Continuous Quality Improvement in Health. Healthcare Policy.

[63] Berwick DM, James B, Coye MJ (2003). Connections between quality measurement and improvement. Medical Care.

[64] Flottorp SA, Jamtvedt G, Gibis B, McKee M (2010). Using audit and feedback to health clinicians to improve the quality and safety of health care. http://www.euro.who.int/__data/assets/pdf_file/0003/124419/e94296.pdf.

[65] Spurgeon P, Mazelan PM, Barwell F (2011). Medical engagement: a crucial underpinning to organizational performance. Health Services Management Research.

[66] Clark J (2012). Medical leadership and engagement: no longer an optional extra. Journal of Health Organization and Management.

[67] Wenger E, McDermott RA, Snyder W (2002). Cultivating Communities of Practice.

[68] The Dartmouth Atlas Working Group Executive Summary of the Dartmouth Atlas of Health Care. http://www.dartmouthatlas.org/pages/executive_summary.

