# Supplementary material for: Bridging the Gap between Theory and Practice in Integrated Care: The Case of the Diabetic Foot Pathway in Tuscany
Source: Int J Integr Care. 2016 May 24;16(2):9. doi: 10.5334/ijic.1991 (PMC5356204; doi:10.5334/ijic.1991)
Supplement: Supplementary file 1 [file ijic-16-2-1991-s1.pdf]

SUPPLEMENTARY FILES

File N.1 PES calculation methods for PES indicator “Diabetes-related lower limb major amputation rate per million residents”

**Definition:**	Diabetes-related lower limbs major amputation rate per million residents
**Numerator:**	No. of Diabetes-related lower limb major amputations
**Denominator:**	Population resident in the LHA area
**Mathematical formula:**	No. of Diabetes-related major amputations at lower limbs *1,000,000<br>---<br>Population resident in the LHA area
**Notes:**	Inpatients admissions of residents in the region are considered, including extra-regional hospitalizations.<br><br>ICD9-CM codes in principal or secondary diagnosis: 250.xx diabetes mellitus<br><br>Surgical procedures ICD9-CM codes<br>84.10 lower limb amputation not otherwise<br>84.12 amputation through foot<br>84.13 disarticulation ankle<br>84.14 amputation ankle through malleoli tibia fibula<br>84.15 amputation below knee<br>84.16 disarticulation knee<br>84.17 amputation above knee<br>84.18 disarticulation hip<br>84.19 abdominopelvic amputation<br><br>Exclusions:<br>- Traumatic amputation ICD9-CM codes: 895.xx, 896.xx, 897.xx;<br>- DRG codes: 370, 371, 372, 373, 374, 375, 376, 377, 378, 379, 380, 381, 382, 383, 384, 385, 386, 387, 388, 389, 390;<br>- Amputations performed by private hospitals non-accredited by the National Health System.
**Source:**	Regional Reporting System – Hospital Discharge Flow
**Reference parameter:**	Regional Average

File N.2 An example of the MeS-Lab PES Dartboard: Tuscany Region Dartboard 2013

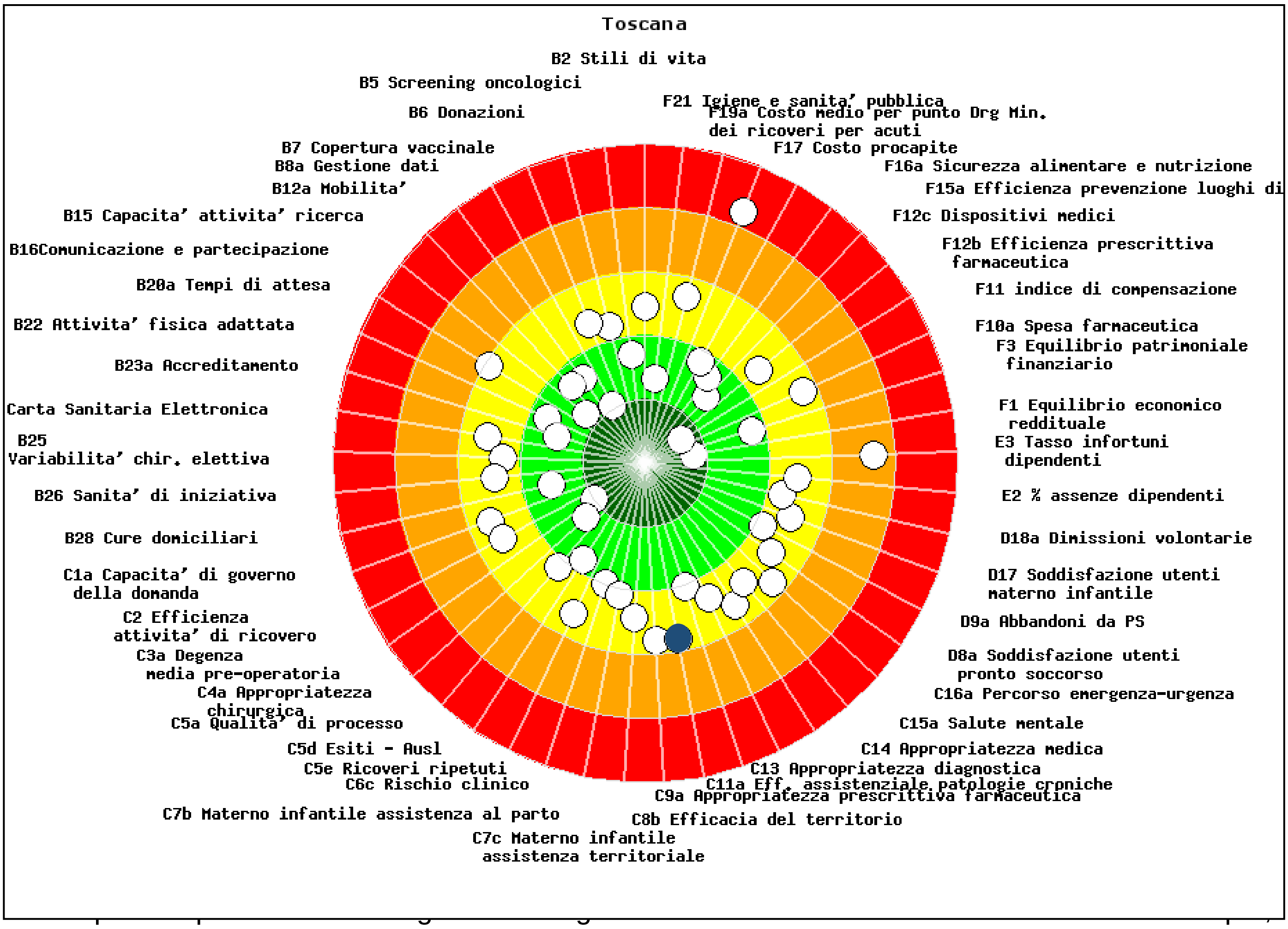

the Diabetes-Related Major Amputation at lower limbs Rate is included in the summary-index for Efficacy in caring for chronic diseases (Diabetes, Heart Failure, COPD, etc) (highlighted in blue).

FILE N.3 CCM Patient Survey Method

**DEFINITION**		Chronic Care Model services provided by the Tuscan Local Health Authorities have been assessed by a survey conducted on the base of the responses into a patient satisfaction survey in 2012.
**REFERENCE POPULATION AND SAMPLING**		The reference population of the survey is represented by Tuscan diabetic and heart failure patients enrolled in 55 out of the 56 of the modules participating in the pilot phase of the Chronic Care Model initiative in Tuscany. Patients affected both by diabetes and heart failure diseases have been excluded from the survey. The sample, randomly selected, is representative of the population of each module and it is stratified according to diabetes and heart failure diseases in each module population. The following eligibility criteria for the inclusion of patients in the survey were applied:<br>(i) patients should have confirmed during the survey interview to behave undergone follow-up visits by a primary care nurse (and by other professionals, such as general practitioners, specialists and healthcare assistants, if present);<br>(ii) patients should have been admitted to the clinic of the reference module at least once in the 12 months preceeding the interview.<br>For each module, there is a statistical significance of 95% and the precision of estimates of 3.5%. For each layer, of the sample different weights were applied according to the reference population.<br>A total of 15,977 patients were contacted for the interviews. 6,606 patients (41% of the total patients contacted) were actually interviewed.
**MEHTODS**		The questionnaire was developed by the MeS-Lab researchers and approved by the Chronic Care Model Regional Commission (CORMAS). The questionnaire, mainly consisting of reporting-type questions, is composed of 58 questions organized in 8 categories:<br>- recruitment (2 questions);<br>- follow-up visits (25 questions). In this section there were the questions on the check-ups performed by nurses such as blood pressure, glycemic values, weight and waist circumference, and feet during follow- up visits;<br>- group meeting (7 questions);<br>- logistic (4 questions) ;<br>- specialist visits (7 questions);<br>- coordination (2 questions);<br>- overall evaluation of the care provided (4 questions)<br>- socio-demographic characteristics of the patients (7 questions).<br>The questionnaire was administered between January and September 2012, with the Computer Assisted Telephone Interviewing (CATI) survey method.

File N.4 “LHA diabetes-related major amputation and revascularization at lower limbs estimated expenditure (total and per 100,000 residents).

LHA	Amputation Costs (Total 2009-2012)	Revascularization Costs (Total 2009-2012)	LHA population (Sum of number of residents 2009-2012)	Amputation Costs (per 100,000 residents) Average 2009-2012	Revascularization Costs (per 100,000 residents) Average 2009-2012	Total Costs (per 100,000 residents) Average 2009-2012
Livorno LHA	€ 694,253.00	€ 2,117,085.00	1,409,700	€ 49,248.28	€ 150,179.83	€ 199,428.11
Prato LHA	€ 444,873.00	€ 1,570,385.95	993,758	€ 44,766.73	€ 158,024.99	€ 202,791.72
Empoli LHA	€ 306,466.00	€ 1,757,176.05	958,528	€ 31,972.57	€ 183,320.26	€ 215,292.83
Pistoia LHA	€ 603,883.00	€ 2,171,785.75	1,168,826	€ 51,665.77	€ 185,809.16	€ 237,474.93
Firenze LHA	€ 1,527,979.00	€ 6,999,834.15	3,279,815	€ 46,587.35	€ 213,421.62	€ 260,008.97
Massa LHA	€ 598,480.00	€ 1,585,541.00	815,142	€ 73,420.34	€ 194,511.02	€ 267,931.36
Viareggio LHA	€ 449,332.05	€ 1,486,483.75	675,958	€ 66,473.37	€ 219,907.71	€ 286,381.08
Lucca LHA	€ 431,587.00	€ 2,166,701.00	894,014	€ 48,275.19	€ 242,356.50	€ 290,631.69
Arezzo LHA	€ 190,083.00	€ 4,377,839.00	1,393,753	€ 13,638.21	€ 314,104.36	€ 327,742.57
Grosseto LHA	€ 811,479.00	€ 2,241,062.00	909,238	€ 89,248.25	€ 246,476.94	€ 335,725.19
Siena LHA	€ 535,249.00	€ 3,261,317.00	1,086,114	€ 49,281.11	€ 300,273.91	€ 349,555.02
Pisa LHA	€ 1,421,539.00	€ 3,394,239.00	1,352,728	€ 105,086.83	€ 250,918.07	€ 356,004.90
